# From Yeast to Therapeutics: Modeling Neurodegenerative Diseases in *Saccharomyces cerevisiae*

**DOI:** 10.1002/yea.70008

**Published:** 2025-11-24

**Authors:** Jose Ribamar Ferreira‐Junior, Vittoria de Lima Camandona, Mario H. Barros

**Affiliations:** ^1^ Escola de Artes, Ciências e Humanidades Universidade de São Paulo São Paulo Brazil; ^2^ Departamento Microbiologia Instituto Ciências Biomédicas, Universidade de São Paulo São Paulo Brazil

**Keywords:** biological process, genetics, molecular biology, molecular neuroscience, physiology

## Abstract

Here, we review the use of *Saccharomyces cerevisiae* as a powerful model organism for studying cellular processes implicated in neurodegenerative disorders, including stress responses, proteostasis impairment, and vesicle trafficking defects. Over the last two decades, baker's yeast models have been developed for complex diseases such as Parkinson's, Alzheimer's, Huntington's, and Amyotrophic lateral sclerosis (ALS). Yeast cells expressing human proteins, such as amyloid‐β, α‐synuclein, huntingtin, and TDP‐43, have become crucial tools for high‐throughput drug screening aimed at counteracting disease progression. These yeast models have unveiled key components involved in the metabolism and toxicity of these proteins, enabling the identification of interacting partners and novel factors within each pathway. Importantly, these pathways were subsequently shown to be conserved in mammalian models. Furthermore, drug candidates identified using yeast models have provided significant leads for drug discovery, highlighting their potential for developing treatments for these neurodegenerative diseases.

## Summary

- Yeast models help find treatments for brain diseases.

## Graphical abstract

Yeast plasmids expressing human Aβ‐42, α‐syn, htt, and TDP‐43. Their fate includes an equilibrium between the endocytic pathway and aggregates that potentially disrupt endocytosis, vesicular transport, vacuolar autophagy, and mitochondrial activity.

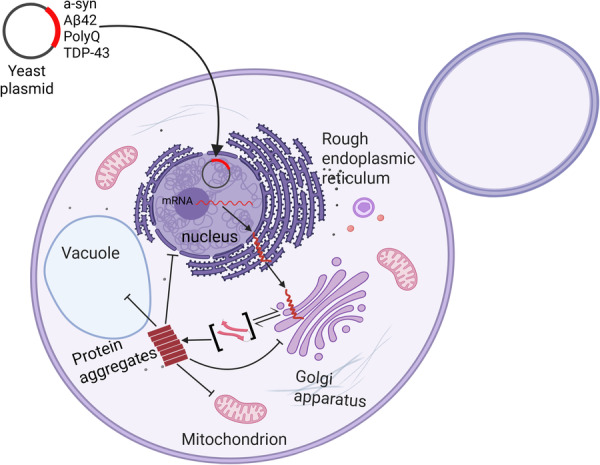

## 1 Introduction

*Saccharomyces cerevisiae* serves as a powerful model for studying neurodegenerative diseases. Although it is impossible to study all the different aspects underlying neurodegenerative diseases in unicellular organisms, factors that cause protein pathologies can be studied in yeast. Basic cell processes that control protein‐folding homeostasis are highly conserved, and the lack of intercellular connections eliminates complexity, providing a focus on the basic protein conformation dynamics.

*S. cerevisiae* biology has been studied for the last 100 years, favored by its simplicity, low cost, and industrial importance in baking and brewing. Researchers have employed yeast as a tool to study every aspect of cell biology. In molecular biology studies, yeast is an unrivaled toolkit; it includes a compact genome, versatile growth as a haploid or diploid, unparalleled genetic facilities, an extensively studied DNA transformation system, and, in the post‐genomic era, the possibility of high‐throughput screenings using comprehensive gene knockout collections. At least a dozen of Nobel Prize‐winning studies were sustained and developed based on the so‐called awesome power of yeast genetics. In the current century, Hartwell's work on key regulators of the cell cycle (2001), Scheckman's in the secretory pathway (2013), and Ohsumi's in the mechanism of autophagy (2016) are among the laureates. Particularly, Scheckman and Ohsumi's seminal works in yeast have been fundamental to understanding the physiology of our cells and the pathophysiology of human diseases, particularly neurodegenerative disorders. Expression of aggregation‐prone proteins disrupts the secretory pathway, directly impairing Golgi function and cellular trafficking. This disruption creates a vicious cycle, as it compromises vacuolar degradation by hindering the delivery of hydrolytic enzymes, thereby preventing the clearance of aggregates via autophagy. Simultaneously, it induces ER stress and the unfolded protein response (UPR). UPR stimulates ER membrane synthesis and regulates the turnover of incorrectly folded proteins by fine‐tuning ER‐associated protein degradation. When the resulting reliance on proteasome degradation fails, cytosolic aggregates accumulate, further exacerbating the initial dysfunction and leading to cell death (Remondelli and Renna 2017).

Building on foundational work by Lindquist et al. *S. cerevisiae* has emerged as a powerful model for studying protein misfolding and trafficking defects in age‐related neurodegenerative disorders (Khurana, Peng, et al. 2017). Notably, yeast prions share molecular properties with amyloidogenic proteins implicated in neurodegenerative diseases (Lindquist 1997). Prions and amyloid‐forming proteins undergo conformational changes that template misfolding onto identical proteins, a process often facilitated by chaperones and remodeling factors such as Hsp104p, involved in their propagation (Lindquist et al. 1998; Du et al. 2019; Ishikawa 2021). Alzheimer's, Parkinson's, Huntington's diseases, amyotrophic lateral sclerosis (ALS), and gliomas have been linked to self‐templating forms of specific amyloid deposits: Amyloid β and tau in Alzheimer's, α‐synuclein in Parkinson's, huntingtin in Huntington's diseases, TDP‐43 in ALS, and GLIPR2 in gliomas. Lindquist's seminal approach involved expressing these human amyloidogenic proteins in yeast, which successfully recapitulated core cytotoxic features observed in the cells of patients (Treusch et al. 2011); yeast cells expressing human Aβ42 (Matlack et al. 2014), α‐synuclein, huntingtin, TDP‐43 (Tardiff et al. 2013), and GLIPR2 (Tardiff et al. 2012) became crucial for unbiased drug search in high‐throughput genetic screenings for these devastating illnesses. In summary, this approach has facilitated both the discovery of aggregate‐induced cellular mechanisms and the screening of chemical libraries for compounds that reverse toxic phenotypes (Ruetenik and Barrientos 2018; Chernoff et al. 2020; Bayandina and Mukha 2023; Stella et al. 2025; Ishikawa 2021; Jiang and MacNeil 2023).

Moreover, the yeast experimental model has been applied to numerous neurological disorders beyond those discussed here, such as mitochondrial neuropathies, Charcot–Marie−Tooth disease, and metabolic and lysosomal storage disorders, as reviewed extensively elsewhere (Franco et al. 2020; Mahmood et al. 2025; González and Hall 2017; Rajakumar et al. 2017; Las Heras et al. 2025). These conditions often arise from defects in fundamental cellular processes such as mitochondrial function, the secretory pathway, autophagy, and the UPR, similar to the modeled diseases highlighted here. Unveiling particular molecular aspects of human neurodegenerative diseases in yeast models depends on genetic and physiological constraints, such as the presence or absence of disease‐gene orthologs in the yeast genome. In this conservative scenario, functional complementation studies promptly determine whether the human disease gene product replaces the function of the yeast gene product. However, in some neurodegenerative diseases, the disease‐associated genes do not have a yeast ortholog, but a gain of function of the disease mutant proteins greatly contributed to pathogenicity and can still be investigated in yeast through heterologous expression (Jiang and MacNeil 2023; Ruetenik and Barrientos 2018; Menezes et al. 2015). Notably, research in the fission yeast *Schizosaccharomyces pombe* model has uncovered that some prion‐like disease‐associated proteins can paradoxically confer cellular protection by indirectly upregulating stress response pathways (Marte et al. 2022).

This review highlights the utility of the yeast model as a first‐line inquiry in neurodegenerative disease research. We discuss how yeast‐based studies have unraveled complex disease mechanisms, facilitated multi‐omic discoveries, and enabled the identification of therapeutic targets for validation in complex models.

## 2 Neurodegenerative Disorders

### 2.1 Parkinson's Disease (PD)

PD is a neurodegenerative disorder that affects predominantly dopamine‐producing neurons. It affects people over 60 years old, but early‐onset occurs in 10%−14% of cases. The main symptoms are tremor, slow movement, muscle stiffness, swallowing problems, speech disorder, and cognitive impairment; as the disease progresses, the symptoms get worse over time. The identification of genetic determinants of PD allowed extensive studies toward the understanding of molecular mechanisms, and yeast models have been fundamental in this process based on the heterologous expression of the human genes SNCA, SNCAIP, PINK1, PARK2, PARK8; or by studying the function and pathological role of the yeast counterparts genes for PARK7, VPS35(PARK17), ATP13A2 (PARK9), and EIF4G1 (PARK18) (Surguchov 2021; Menezes et al. 2015). SNCA expression and α‐synuclein aggregation will be better detailed after a summary of other PD genetic determinants.

### 2.2 PD Genetic Determinants

**Figure 1:**
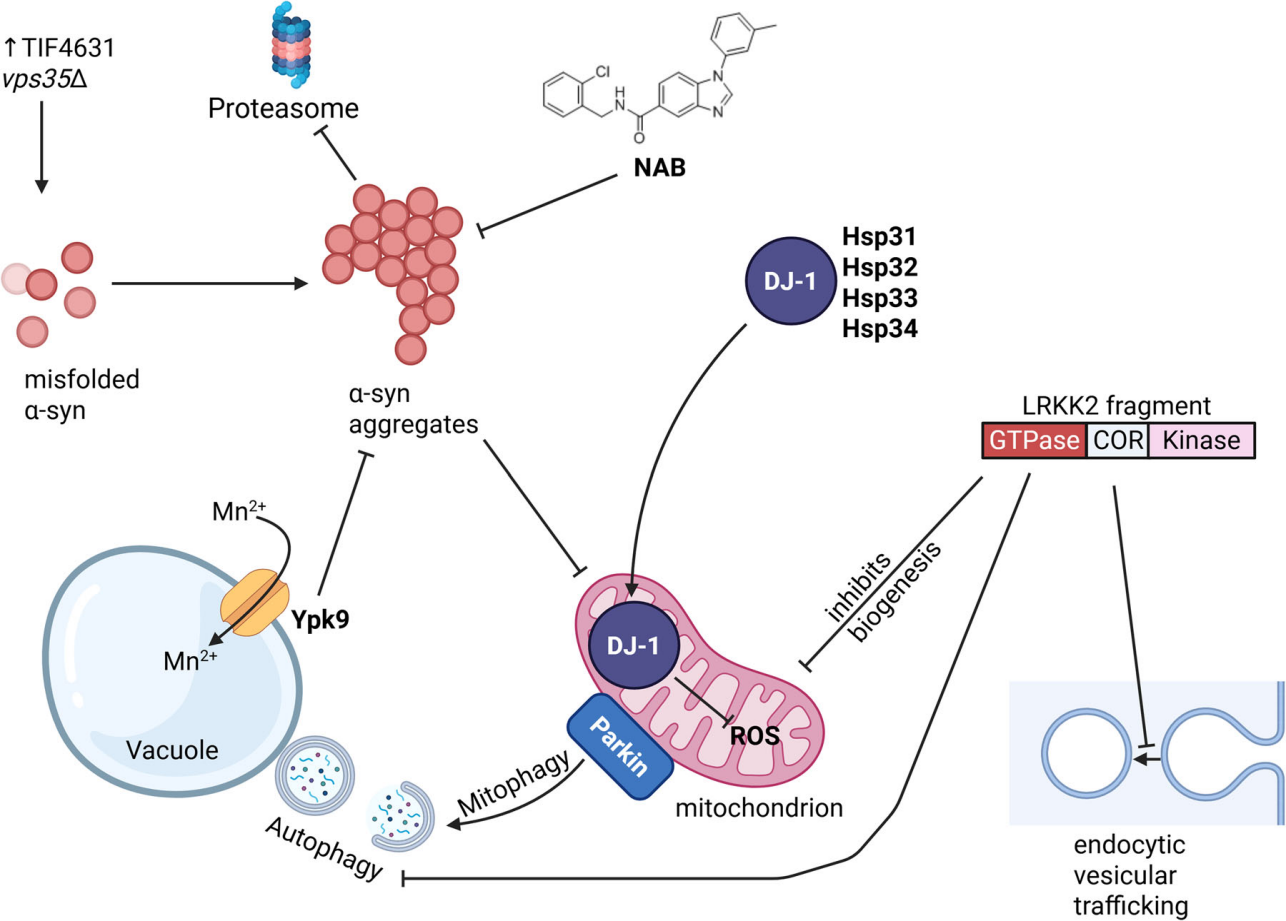
Yeast as a model of Parkinson's disease and intracellular proteostasis. Yeast plasmids expressing α‐synuclein and LRRK2 can be incorporated into the yeast genome. The fate of α‐synuclein depends on its expression level and the quality of the endocytic pathway. Abnormal α‐synuclein accumulation may occur in the cytosol. When the proteostasis is lost, large aggregates of α‐synuclein are formed, disrupting endocytosis, vesicular transport, vacuolar autophagy, and mitochondrial activity. DJ‐1 and related proteins in yeast counteract α‐synuclein‐induced cytotoxicity by lowering mitochondrial superoxide. LRRK2 expression causes disruption of the secretory pathway and blocks mitochondrial function, while Parkin promotes mitophagy.

PARK7 codes for the multifunctional protein DJ‐1, which in yeast has four conserved homologs: Hsp31, Hsp32, Hsp33, and Hsp34. DJ‐1 inhibits α‐synuclein aggregation, and in yeast, these proteins are needed for diauxic‐shift reprogramming and cell survival in the stationary phase (Miller‐Fleming et al. 2014), as well as to promote resistance to several stresses (Natkańska et al. 2017). Upon exposure to redox stress, Hsp31p translocates to the mitochondria, where DJ‐1 paralogs regulate mitochondrial dynamics and ROS homeostasis (Bankapalli et al. 2020). Overexpression of DJ‐1 or yeast counterparts reversed α‐synuclein‐dependent cellular toxicity, and functionally complemented Hsp31, suppressing mitochondrial superoxide levels (Figure 1) (Zondler et al. 2014; Bankapalli et al. 2015). VPS35 (PARK17) has been implicated in familial cases of PD (Menezes et al. 2015). As discussed later, DJ‐1 is also important in preventing oligomerization of Aβ‐42, tau, HTT, and TDP‐43 (Sajjad et al. 2014; Jana et al. 2023; Jimenez‐Harrison et al. 2023).

EIF4G1 (PARK18) is an autosomal dominant PD determinant gene and has two homologs in yeast: TIF4631 and TIF4632, which encode the translation initiation factor eIF4G. Upregulation of eIF4G causes defects associated with protein misfolding (Dhungel et al. 2023). Yeast synthetic lethal screen for TIF4631 and VPS35 converged to a complex of proteins important in recycling transmembrane receptors from endosomes to the trans‐Golgi network consisting of Vps26, Vps35, Vps29, Vps5, and Vps17—the retromer, which orchestrates the intracellular sorting of thousands of integral membrane proteins and, therefore, essential for α‐synuclein toxicity (Dhungel et al. 2023; Simonetti and Cullen 2018). Indeed, early studies in yeast demonstrated that Vps35p participates in vesicle trafficking modulating α‐synuclein cytotoxicity (Tardiff et al. 2013). By recycling these essential receptors, the retromer ensures that the Golgi apparatus remains a fully functional station for processing and sorting.

ATP13A2 (PARK9) encodes for lysosomal P‐type ATPase, while the yeast counterpart YPK9 encodes a vacuolar transporter with a possible role in sequestering divalent heavy metals, which are assumed environmental risk factors for PD; yeast YPK9 protects cells from manganese toxicity and α‐synuclein cytotoxicity. Gitler et al. demonstrated that the protective role of Ypk9 against α‐synuclein toxicity is conserved across species, and that overexpression of the mammalian PARK9 gene can mitigate α‐synuclein‐induced neurodegeneration in various animal models (Gitler et al. 2009; Yeger‐Lotem et al. 2009).

PARK8 encodes for LRKK2 (leucine‐rich repeat kinase 2), whose mutations are the most frequent genetic cause of PD. This gene has no counterpart in yeast; however, LRRK2 heterologous expression leads to growth arrest, and the separate expression of the LRRK2 GTPase domain is toxic, resulting in impairments in endocytic vesicular trafficking, autophagy, and mitochondrial biogenesis in aging yeast (Xiong et al. 2010; Aufschnaiter et al. 2018).

Finally, PARK2 encodes the protein Parkin, a 465‐residue E3 ubiquitin ligase with a particular affinity for proteins in the mitochondrial outer membrane, where it, together with Pink1, controls organelle quality by mitophagy (Seirafi et al. 2015). Parkin has no yeast homolog, but human Parkin expressed in yeast is also directed to mitochondria under oxidative stress, whereas it promotes mitophagy and extends life span (Pereira et al. 2015).

### 2.3 α‐Synucleinopathies

Lewy body formation, driven by α‐synuclein aggregation, is the pathological hallmark of α‐synucleinopathies, including PD. Abnormal accumulation of α‐synuclein leads to protein misfolding and aggregation, damaging multiple cellular structures and ultimately causing cell death, a central mechanism in disease pathogenesis. α‐synuclein is a 140‐residue amphipathic protein encoded by the *SNCA* gene. Primarily expressed in the cerebral cortex system, it exhibits a strong affinity for phospholipids in cellular membranes (Jakes et al. 1994). The N‐terminus of α‐synuclein contains KTKEGV repeats that mediate interactions with membrane phospholipids, promoting a shift from random coil to an α‐helical conformation, and its central region harbors the hydrophobic NAC domain, essential for fibrillization (Giasson et al. 2001). Consequently, α‐synuclein can adopt an amphiphilic helix that binds the surface of synaptic vesicles. However, at high concentrations, membrane‐bound α‐synuclein can disrupt lipid membranes or assemble into β‐sheet‐rich aggregates. These prion‐like aggregates propagate misfolding and ultimately form Lewy bodies, leading to neuronal cell death (Dasari et al. 2022). Yeast models expressing human *SNCA* represent the most extensively studied system for PD. In yeast, α‐synuclein recapitulates key features observed in human cells: it traffics via the secretory pathway to the plasma membrane, exhibits dose‐dependent gain‐of‐function toxicity, and aggregates. Early studies demonstrated that the expression of one or two copies of *SNCA* under the control of a GAL promoter does not affect cell viability; α‐synuclein folds correctly and localizes to the membrane. The expression of three copies is sufficient for α‐synuclein aggregation, which affects growth. With four copies, the high toxicity of the resulting aggregates leads to cell death. These aggregates in yeast are clustered in vesicle structures reminiscent of the Lewy bodies (Outeiro and Lindquist 2003). Yeast α‐synuclein aggregation and vesicle accumulation lead to diverse cellular stressors. These include increased lipid droplet formation, proteasome inhibition, focal bursts of nitric oxide, ROS and protein nitration, mitochondrial dysfunction, and disrupted metal ion homeostasis; mitochondrial dysfunction is exacerbated in the presence of oxidants, such as menadione, which can be mitigated by melatonin addition and mitophagy activation (Zampol and Barros 2018). Yeast studies reveal that α‐synuclein exists in a dynamic equilibrium; altered protein levels content unbalance the equilibrium, recruiting functional membrane proteins into toxic cytoplasmic inclusions (Figure 1) (Outeiro and Lindquist 2003). Crucially, a study demonstrated that even minimal expression of human α‐synuclein induces the formation of respiration‐deficient rho‐*petites* mutants. Strikingly, α‐synuclein is imported into mitochondria in yeast (X. Zhang et al. 2024), and full α‐synuclein expression in petite cells triggers apoptosis, indicating that mitochondrial respiration is not absolutely required for α‐synuclein‐mediated cell death (Akintade and Chaudhuri 2020). This finding provides new insights into PD pathogenesis, suggesting that mitochondrial impairment, rather than complete function, may be crucial in α‐synuclein toxicity. Notably, mutant forms of α‐synuclein (A53T and A30P) did not induce petite formation, highlighting distinct mechanisms for wild‐type (WT) toxicity. These findings support the hypothesis that early mitochondrial dysfunction contributes to neurodegeneration in PD. Finally, α‐synuclein can also be found in the yeast nucleus. The nucleolar factor Dbp4, a DEAD‐box helicase, stabilizes a fraction of α‐synuclein oligomeric species, while its downregulation decreases α‐synuclein toxicity, and therefore the human ortholog DDX10 has emerged as a possible drug target (Popova, Wang, Rajavel, et al. 2021). The pathogenic mechanism of mitochondrial protein import clogging has been comprehensively reviewed recently (Coyne et al. 2023).

### 2.4 The Yeast Model of α‐Synucleinopathies

The relevance of α‐synuclein segments in proper membrane localization was also investigated in yeast. Accordingly, the expression of an N‐terminus‐truncated version of α‐synuclein leads to the loss of membrane localization and detection inside the mitochondria; likewise, the expression of the truncated version of α‐synuclein at the NAC domain reduces cytosolic accumulation (Soper et al. 2008; X. Zhang et al. 2024). Not only can the WT α‐synuclein lead to PD in humans, but the point mutant alleles A53T and A30P are also associated with rare forms of early‐onset familial PD and accelerate α‐synuclein aggregation events (Narhi et al. 1999). The excess of the WT, or A53T variant, impaired the secretory pathway at early stages, leading to vesicle accumulation and α‐synuclein aggregation. Differently, the A30T variant showed reduced binding to membranes, failed to enter the secretory pathway, and was less toxic to neurons and yeast cells (Dixon et al. 2005; Outeiro and Lindquist 2003). Using the α‐synuclein yeast expression system, it was possible to build a mutant library of 2600 possible α‐synuclein single‐point mutants that exhaustively sample substitutions of each amino acid (aa) at every position, providing conditions to identify substitutions that disrupt the toxic membrane‐binding region (Newberry et al. 2020). Cellular stresses relevant to α‐synuclein proteotoxicity have also been investigated in yeast cells, such as perturbations to vesicle trafficking and the endosomal network, oxidative stress, the UPR, calcineurin stimulation, protein degradation and autophagy, chaperone activity, and membrane composition (Shadrina and Slominsky 2021; Menezes et al. 2015; Popova, Wang, Rajavel, et al. 2021). Cells overexpressing calcineurin B displayed an enhanced localization of A53T variant to the cytosolic membrane and lower aggregation, while proteasome inhibition by α‐synuclein depends on yeast Rpn1 and the human counterpart PAAF1 (Chawla et al. 2023; Galka et al. 2024). Environmental inducers of synucleinopathies were also investigated using yeast, with the study of pesticides cymoxanil and metalaxyl promoting aggregation of α‐synuclein (Amaral et al. 2024).

### 2.5 α‐Synuclein Detox Screens

Yeast genome‐wide studies systematically screen the entire genome to identify every gene that modifies proteotoxicity when overexpressed or deleted. Using only the deletion collection, 86 gene mutants were shown to be more sensitive to α‐synuclein (Willingham et al. 2003); however, combined tests of the mutant deletion collection with a pool of overexpression strains show a total of 332 genetic modifiers of α‐synuclein toxicity. Another overexpression study identified 54 suppressors and 23 enhancers of α‐synuclein toxicity (Yeger‐Lotem et al. 2009). In these studies, most genes clustered in processes related to a calcium signaling hub, also linked to perturbed mitochondrial quality control and function (Khurana, Chung, et al. 2017). With the toxicity of α‐synuclein well established in yeast and consistent with the proteotoxicity observed in neurons, experiments were designed to find compounds and drug candidates that could halt the damage caused by α‐synuclein‐mediated toxicity. High‐throughput screening of commercial chemical libraries in α‐synuclein‐expressing yeast identified bioactive compounds counteracting its toxicity. Initial screens highlighted the efficacy of 8‐hydroxyquinoline metal chelators (Tardiff et al. 2012). Subsequent work identified N‐aryl benzimidazole (NAB) as a more potent suppressor (Figure 1). The mechanism of action of NAB was indeed related to α‐synuclein toxicity; with the identification of genetic changes that restore NAB target function, it was possible to verify that NAB promoted endosomal transport events disrupted by α‐synuclein aggregates, that is, those dependent on the E3 ubiquitin ligase Rsp5p/Nedd4 (Tardiff et al. 2013). After the identification of this druggable pathway in yeast, it was verified that NAB also protects cortical neurons derived from a patient with PD, indicating that NAB engagement with Rsp5p/Nedd4 at its N‐terminus improves endosomal transport and restores vesicle trafficking affected by α‐synuclein cytotoxicity (Hatstat et al. 2021). The α‐synuclein toxicity in yeast also led to the identification of peptides and different drugs (AGK2, XCT790, phloretin, Cpd9) that counteract its aggregation and are candidates for the development of future therapeutic interventions (Ali et al. 2025; Popova, Wang, Pätz, et al. 2021; Suresh and Manjithaya 2019; Du et al. 2019). The flavonoid baicalein, known for its neuroprotective effects including the inhibition of α‐synuclein aggregation and oligomer disaggregation in mammalian models (Y.‐H. Wang, Yu, et al. 2013; Hu et al. 2016), significantly reduced α‐synuclein toxicity in the sensitive yeast strain TK01. Remarkably, baicalein prevented cytotoxicity at concentrations lower than those effective in vitro, demonstrating the model's enhanced sensitivity for detection (Zhu et al. 2004; Sangkaew et al. 2022). This validated baicalein's neuroprotective potential underscores the utility of yeast for discovering therapeutic candidates against neurodegeneration.

## 3 Alzheimer's Disease (AD)

AD is the most common chronic neurodegenerative disease. AD is a progressive and irreversible disorder that causes memory loss. The progressive cognitive and behavioral symptoms that characterize AD are elicited from functional changes observed in brain cells. While its origin is multifactorial, it is defined at the neuropathological molecular level by amyloid plaques and neurofibrillary tangles. Amyloid‐β (Aβ) aggregation possibly leads to a cascade of pathogenic processes, such as inflammation, neurofibrillary tau‐tangle formation, synapse dysfunction, neuronal death, and cognitive impairment. The amyloid precursor protein (APP) is an integral membrane protein expressed in many tissues and concentrated in neurons; its role has been implicated as a regulator of synapse formation (Priller et al. 2006). Aβ peptides of 40 aa long are proteolysis products of APP by α and γ‐secretases, whereas those of 42 aa are products of APP by β‐ and γ‐secretases. APP is processed by β‐secretase in the extracellular space to produce a membrane‐tethered fragment known as C99, which undergoes sequential cleavages by γ‐secretase, generating a series of β‐amyloid peptides. β‐ and γ‐secretase activities are located in the endosomal compartment and trans‐Golgi network; therefore, Aβ is generated in these subcellular localizations and subsequently secreted through exocytosis. The Aβ 42‐aa peptide is considered more toxic and more prone to produce oligomeric species than the Aβ 40‐aa peptide (Jarrett et al. 1993). Aβ exists as monomers, dimers, higher oligomers, amyloid polymers, and amyloid fibrils, and forms the amyloid plaque.

### 3.1 AD Genetic Determinants

Genetic determinants for early‐onset have been described in four genes: APP, PSEN1 (presenilin 1), PSEN2 (presenilin 2), and SORL1 (Sortilin‐Related Receptor 1). Presenilin 1 and 2 are components of the γ‐secretase complex; SORL1 encodes a neuronal sorting receptor involved in trafficking APP away from amyloidogenic pathways; mutations in these have high or complete penetrances to develop early‐onset AD (Escamilla‐Ayala et al. 2020; Jensen et al. 2023). For late‐onset AD, the greatest genetic risk factor is the apolipoprotein ApoE. APOE encodes for three isoforms of a secreted 299 aa protein: apoE2, apoE3, and apoE4. One APOE‐ε4 allele raises the risk of developing AD three times, and the homozygous APOE‐ε4/APOE‐ε4 condition has an eightfold risk (DiBattista et al. 2016). In the cellular neuropathology of AD, and other dementia processes, it is notable the formation neurofibrillary tangles resultant of hyperphosphorylated tau protein aggregates; its appearances can be caused by Aβ oligomerization and tau mutations (Oddo et al. 2003); tau proteins are a group of six highly soluble protein isoforms produced by MAPT gene alternative splicing; it is highly abundant in neurons with a direct role in axon microtubules stabilization. tau is phosphorylated by kinases such as GSK3α, GSK3β, MAPK13, and AMP‐activated protein kinase; aberrant hyperphosphorylation occurs on several epitopes (Thr181, Thr231, Ser202, Ser205, Ser214, Ser396, Ser404, Ser409, and Ser422) (Trinczek et al. 1995).

### 3.2 Yeast Models of Tau Toxicity

**Figure 2:**
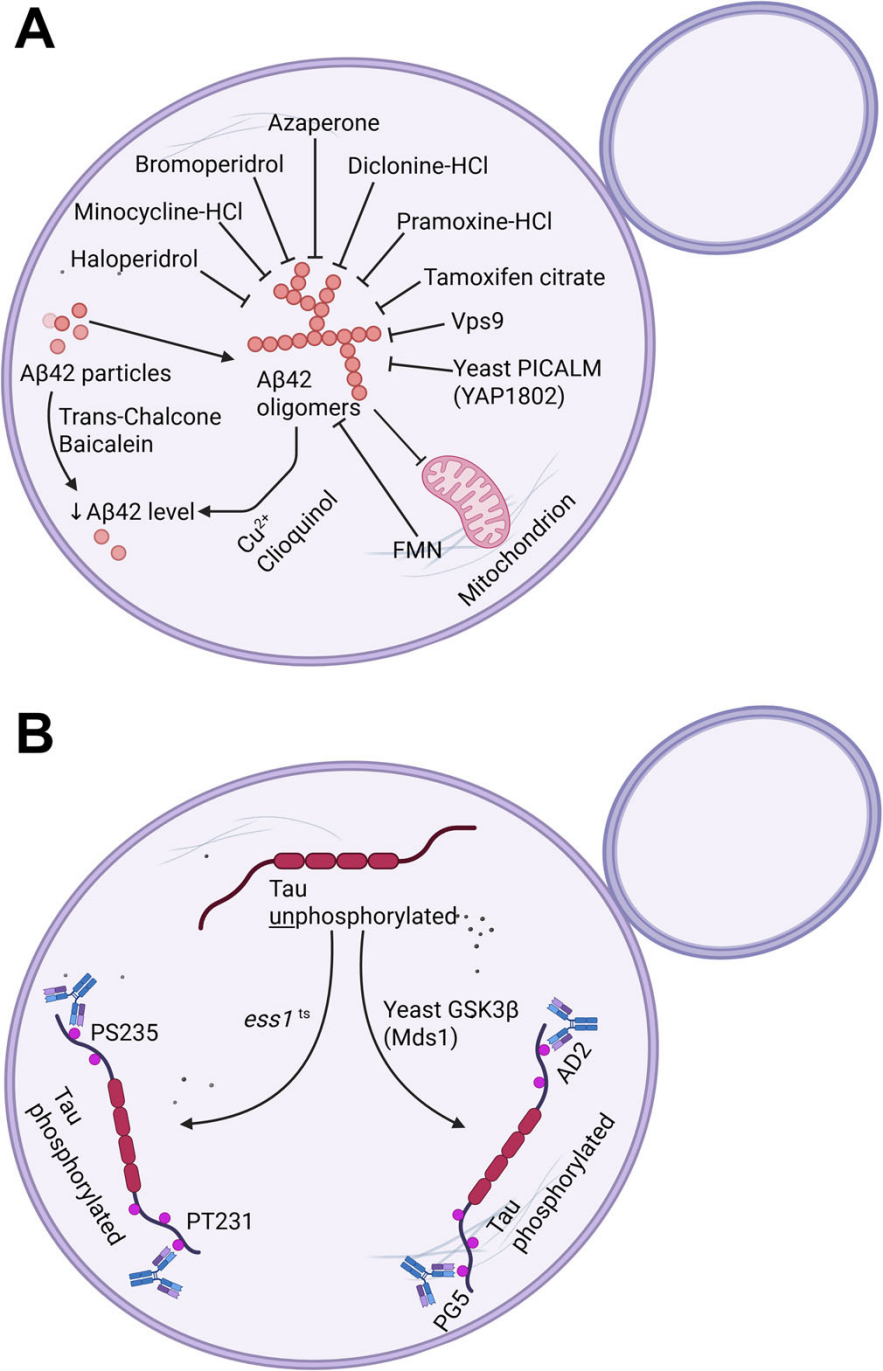
Yeast as a model of Alzheimer's disease. (A) Compounds and proteins (Vps9 and Yap1802) that modify the citoxicity of Aβ42 in yeast. (B) Yeast proteins involved in Tau phosphorylation. Mds1 is an orthologue of mammalian GSK3β. Ess1p is the yeast ortholog of human Pin1, and decreased Pin1 expression/activity is reported in cases of Alzheimer's disease. As *ESS1* is an essential gene, depletion of its protein activity is obtained using a *ts* mutant.

Studies with yeast models of tau toxicity have elucidated key aspects of tau phosphorylation and aggregation using conformation‐dependent antibodies to detect pathological filament formation (De Vos et al. 2011; Vandebroek et al. 2005). Nevertheless, human tau protein was not found to aggregate in yeast (Zubčić et al. 2024). Yeast expresses functional orthologs of major human tau kinases: Mds1 corresponds to GSK3β, and Pho85 corresponds to CDK5. Intriguingly, *pho85* mutants exhibited tau hyperphosphorylation at the AD2 and PG5 epitopes, mirroring the hyperphosphorylation observed upon CDK5 depletion in human systems with an inhibition of inositol phosphate and sphingolipid pathways (Randez‐Gil et al. 2020); glucose starvation also led to increased phosphorylation of tau protein in fission yeast (Yılmazer et al. 2025). Further investigation in yeast demonstrated distinct roles for these conserved kinases, showing that Mds1/GSK‐3β acts genetically downstream of Pho85/CDK5. This study also revealed that oxidative stress triggers tau aggregation via a mechanism parallel to hyperphosphorylation (Vanhelmont et al. 2010). Additionally, the function of the prolyl isomerase Pin1 (yeast Ess1) was examined in yeast. Ess1 isomerizes phosphorylated tau, preventing or reversing filament formation, while its depletion resulted in tau hyperphosphorylation and impaired growth (Figure 2) (De Vos et al. 2011). The synergic effect of tau and α‐synuclein co‐expression in yeast was investigated, with an increment of tau insolubility correlated with increased tau phosphorylation in S396/404 (Ciaccioli et al. 2013).

### 3.3 Aβ Citoxicity in Yeast

Yeast models have also been developed to investigate Aβ citoxicity (Dhakal and Macreadie 2020). Early yeast studies were focused on the expression of the human APP protein and the identification of enzymes with secretase activity (Hines et al. 1994; H. Zhang et al. 1994). The γ‐secretase was reconstituted in yeast by the co‐expression of human PS1, Nct, APH‐1, and PEN‐2 genes, and its biological activity was assessed (Yonemura et al. 2011). Using an APP like reporter, this yeast heterologous system has been used to evaluate the pathogenicity of PS mutations found in AD patients (Imai et al. 2019). With the direct expression of Aβ peptides in yeast cytosol, it was possible to investigate the consequences of Aβ accumulation, such as the heat shock protein response (Caine et al. 2007), and the initial stages of Aβ oligomerization (Bagriantsev and Liebman 2006). Aβ toxic oligomers indicate a protein‐folding problem associated with other neurodegenerative diseases and with yeast prions (Shorter and Lindquist 2004; Winderickx et al. 2008). The proper address of Aβ to the yeast reticulum and the secretory pathway mimicked the multiple compartments distribution of Aβ through the secretory pathway (Treusch et al. 2011). Addressing Aβ to the reticulum resulted in a lower growth rate, lower biomass yield, a lower respiratory rate, and increased oxidative stress (Chen and Petranovic 2015; Treusch et al. 2011). Indeed, intracellular amyloid β (iAβ) is a strong player in neurodegeneration (Gallego Villarejo et al. 2022).

### 3.4 Modifiers of Aβ Citoxicity in Yeast

The identification of phenotypes caused by Aβ toxicity in yeast allowed genetic screens for modifiers such as suppressors or enhancers. Notably, a suppressor screen revealed that overexpression of *VPS9* and other endocytic genes rescues Aβ toxicity. This demonstrated that endocytosis, particularly clathrin‐mediated endocytosis (CME), is a critical point of vulnerability to Aβ. The core machinery of CME (clathrin, dynamin, adapters) is highly conserved from yeast to humans, and it facilitates the internalization and recycling of receptors involved in diverse cellular processes (Treusch et al. 2011). Strikingly, several yeast genes identified as modifiers of Aβ toxicity encode homologs or functional partners of established human AD risk factors. These include PICALM (phosphatidylinositol binding clathrin assembly protein), BIN1, and CD2AP—all essential for CME in both yeast and humans (Figure 2). This conserved requirement strongly suggests that Aβ accumulation impairs the CME pathway. Overexpression of the yeast ortholog of PICALM reduced oligomerization of Aβ; likewise, a drug screen identified seven compounds that reduce the oligomerization of the cytosolic form in yeast: bromperidol, haloperidol, azaperone, pramoxine HCl and dyclonine HCl, tamoxifen citrate, and minocycline HCl (Park et al. 2016). Using a yeast Aβ reticulum‐addressed model, a genetic unbiased screen of 140,000 small compounds identified 8‐hydroxyquinolines that partially rescue yeast cells from Aβ toxicity (Matlack et al. 2014); 8‐hydroxyquinolines were similarly identified in earlier yeast screens using Aβ, TDP‐43, and α‐synuclein (Tardiff et al. 2012). Clioquinol is an 8‐hydroxyquinoline that showed effective rescue of the yeast endocytosis pathway by promoting metal‐dependent Aβ oligomers degradation within the secretory and endosomal compartments (Matlack et al. 2014). Synergic studies were also conducted in yeast. Trans‐chalcone plus baicalein reduced intracellular Aβ42 (Dhakal et al. 2021). Interestingly, a synthetic genetic array (SGA) of yeast collection mutants with the yeast Aβ model identified among the affected genes FMN1, which encodes for riboflavin kinase necessary for flavin mononucleotide (FMN) production. In this study, the supplementation with FMN showed protection against Aβ oligomerization and improved redox homeostasis of the yeast cells (Chen et al. 2020).

## 4 Huntington's Disease (HD)

HD is a progressive neurological disorder whose triad of symptoms is motor dysfunction, cognitive impairment, and neuropsychiatric features. Involuntary movement disorder (chorea), voluntary movement impairment and bradykinesia are observed in the motor dysfunction component of the disease (Reilmann et al. 2014); problems of attention, mental flexibility, planning, emotion recognition, and difficulty in speech may lead to social disengagement and decreased participation in conversations, and are collectively some of the cognitive impairment symptoms and, finally, irritability, depression, and apathy are observed as behavioral features (Papoutsi et al. 2014). Besides these symptoms, the mood changes in the patients are fairly dramatic, which makes the situation more challenging for the families affected. HD is caused by a CAG expansion in the exon 1 of the Huntingtin gene (HTT) on chromosome 4, inherited in an autosomal dominant pattern. The CAG triplet encodes the aa glutamine, and its expansion forms a polyglutamine (polyQ) tract at the N‐terminus of HTT, involved in the formation of oligomers and aggregates that are cytotoxic (DiFiglia et al. 1997). In typical individuals, the length of the CAG repeats is 16–20 on average, whereas in affected patients, this expands to over 36 units. The number of CAG repeats in the mutant HTT allele is inversely correlated with the age at which the first diagnosable symptoms manifest; specifically, adult‐onset cases have 40–50 repeats, while more than 60 are observed in juvenile HD (Lee et al. 2022; Langbehn et al. 2004).

HTT aberrant splicing results in the production of a transcript containing only exon 1, expressing 17 aa of the N‐terminal part, followed by the polyQ tract, and a proline‐rich domain, which is prone to aggregation inside the cell in disease (Bates et al. 2015). Similarly, proteases such as caspases and calpains cleave full‐length HTT and generate an N‐terminal fragment with a comparable organization (Graham et al. 2006; Gafni et al. 2004). As the concentration of this fragment builds up, it forms highly ordered β‐sheet amyloid fibril aggregates that may sequester protein quality control factors in the cytoplasm or transcription factors inside the nucleus, and disrupt the cell's activities (Jayaraman et al. 2012). Below, we describe works that used the HTT N‐terminal fragment and its variants to model HD protein aggregation in yeast; to understand amyloid fibrils contribution to the loss of function observed in the cell, to track proteins and organelles affected, and genes that either augment or diminish these phenotypes.

### 4.1 PolyQ Expression in Yeast

**Figure 3:**
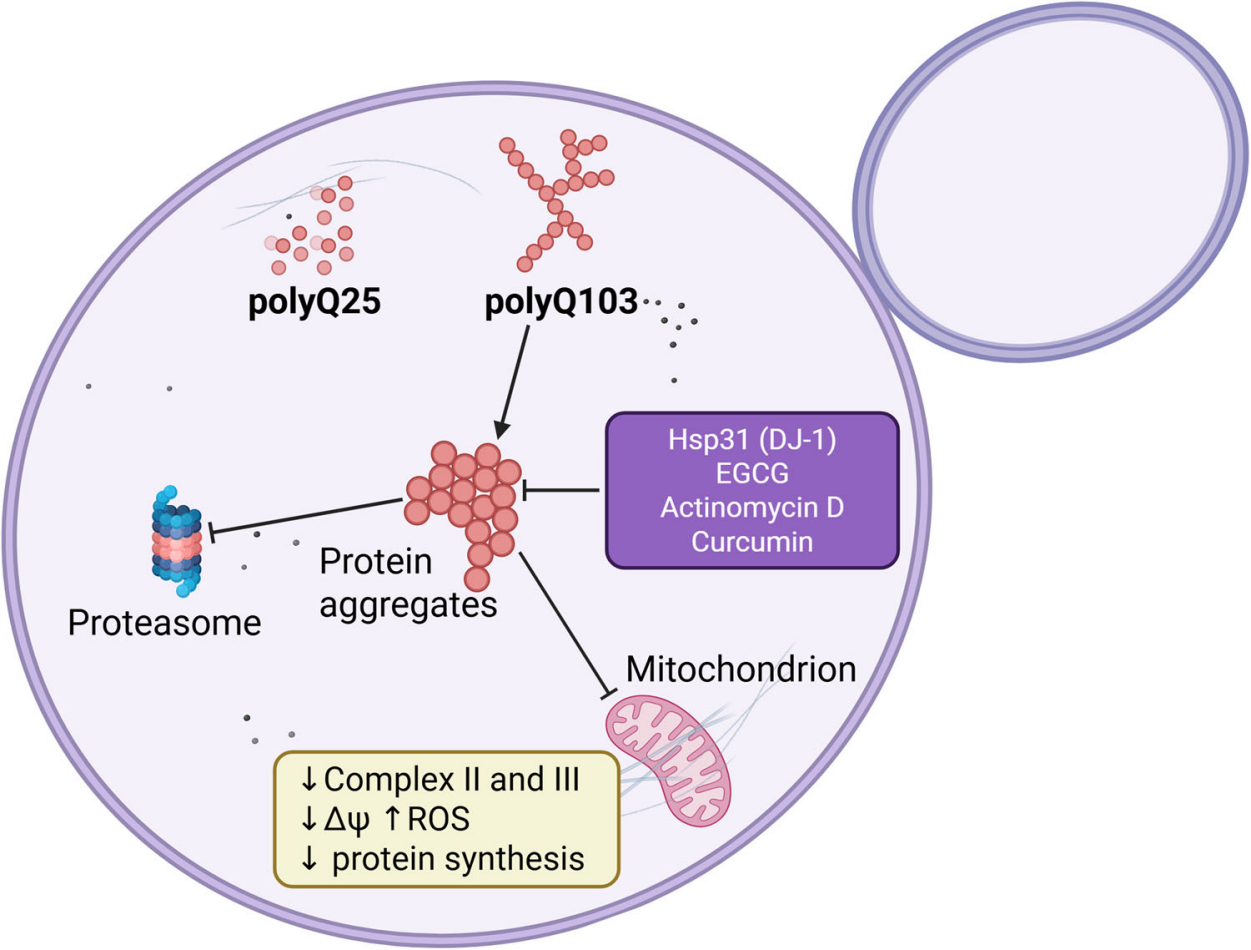
Yeast as a model of Huntington's disease: In yeast models of Huntington's disease, expression of huntingtin fragments with pathogenic (Q103) versus non‐pathogenic (Q25) polyglutamine lengths reveals distinct fates. While the Q25 fragment remains soluble, the Q103 fragment forms aggregates. These aggregates disrupt cellular proteostasis and impair mitochondrial function, specifically reducing the activity of respiratory complexes II and III. This mitochondrial dysfunction is further characterized by a decreased membrane potential and diminished mitochondrial protein synthesis. Notably, certain molecular factors, such as Hsp31 and drugs, can mitigate the detrimental effects of these aggregates.

The standard approach for the yeast model of HD involves expressing the HTT exon 1 fused with GFP that allows the observation of protein aggregates by fluorescence microscopy. Besides, polyacrylamide gel electrophoresis (SDS‐PAGE) followed by western blot analsyis is usually performed to verify the distribution of soluble and insoluble polyQ proteins. In this model, the expression of different N‐terminal fragments of HTT containing 25, 47, 72, or 103 glutamine residues shows a polyQ length dependence on protein aggregation abundance (Dehay and Bertolotti 2006; Krobitsch and Lindquist 2000). The Q25 tract stays soluble and does not show any aggregation, but cells expressing Q103 contain most of the fusion protein insoluble in large aggregates (Krobitsch and Lindquist 2000; Meriin et al. 2002). Interestingly, the deletion of the chaperone HSP104 abrogates the formation of aggregates completely, but its overexpression increases the number of fluorescent foci, indicating a protein folding control on the formation and dissolution of aggregates (Krobitsch and Lindquist 2000). Moreover, the proline‐rich region of HTT exon 1 is a critical determinant for aggregation; indeed, this segment deletion strongly alters Q103 aggregate formation pattern (Dehay and Bertolotti 2006). When expressed in yeast, Q103 alters mitochondrial function; it reduces oxygen consumption and diminishes complexes II and III activities of the mitochondrial respiratory chain (Solans et al. 2006), which also leads to elevated levels of ROS (Figure 3). A direct association between the polyQ tract and the mitochondrial outer membrane dissipates the mitochondrial membrane potential, decreases energetic coupling, and impairs mitochondrial protein synthesis (Ocampo et al. 2010). This toxicity is reduced by the overexpression of HAP4, a central regulator of the transcription of many nuclear genes encoding mitochondrial proteins; Hap4p rescues partially most of the aforementioned phenotypes, although polyQ aggregate foci are at the same level as those in control cells (Ocampo et al. 2010). Similarly, in multicellular organisms, PGC‐1α is a transcriptional activator that regulates mitochondrial biogenesis, and its excess in *Drosophila melanogaster* also attenuates polyQ‐induced toxicity, indicating a conserved mechanism and a possible intervention for HD treatment by modulation of PGC‐1α levels (Ruetenik et al. 2016; Q. Zhang et al. 2019; D'Egidio et al.^2025^). Integrated multi‐omic analysis of Huntington disease and yeast model shows deregulated pathways that are common to human, mice, and yeast systems, including metabolism of various aa, glutathione metabolism, autophagy, mitophagy, and cell proteostasis (Pradhan et al. 2022; Takaine et al. 2022).

PolyQ aggregates were studied in yeast in situ by cryoelectron tomography at a nanoscale resolution; the 3D images allowed the observation of unstructured inclusion bodies and less frequent fibrillar aggregates, in contrast to mammalian cells, where polyQ aggregates are exclusively fibrillar (Gruber et al. 2018). Cryoelectron tomography further reveals significant alterations in mitochondrial morphology. These changes occur without direct physical interaction with inclusions or fibrils and may arise from polyQ proteins inserted into the mitochondrial outer membrane (Ocampo et al. 2010). Fluorescence resonance energy transfer‐based method (FRET) can also be used to monitor polyQ aggregation in individual yeast cells (Wan et al. 2022).

As previously mentioned in the PD section, Hsp31p is the *S. cerevisiae* ortholog of human DJ‐1 (PARK7), and a member of DJ‐1/ThiJ/PfpI superfamily (Aslam and Hazbun 2016) that translocates from the cytosol to the mitochondria upon redox challenge, and protects against reactive oxygen species (Bankapalli et al. 2015). Similarly, in the context of α‐synuclein cytotoxicity, Hsp31p overexpression increases soluble Q72 levels, and this prevents aggregation of the huntingtin N‐terminal fragment and suppresses polyQ toxicity (Sajjad et al. 2014). Besides its effect on ROS production, whether Hsp31 affects polyQ association or has an effect on the outer mitochondrial membrane is yet to be explored.

### 4.2 Poly Q Detox Screening

Screens for natural compounds or small molecules using the yeast model system offer a feasible approach to discover potential drug candidates for HD treatment. For instance, the component of green tea (‐)‐epigallocatechin‐3‐gallate (EGCG) inhibits polyQ aggregation in both yeast and fly models of HD (Ehrnhoefer et al. 2006; Varga et al. 2020). EGCG likely suppresses the formation of small polyQ oligomers, leading to the formation of larger ones, which may aid the cell in sequestering the mutant protein and preventing disruption of its organelles' function. A library containing ~11,000 natural product extracts from bacterial and fungal sources, isolated from Papua New Guinea, Costa Rica, and other locations, yielded four extracts that improve, by at least 30%, the growth of cells expressing Pro‐Q103 (huntingtin toxic mutant that lacks the proline‐rich domain) (Walter et al. 2014). Bioactivity‐guided isolation of the active molecule found dactinomycin (actinomycin D), a transcription inhibitor, as the active part (Goldberg and Rabinowitz 1962; Walter et al. 2014). In addition to suppressing cell growth, dactinomycin inhibits Q103 aggregation within a low concentration range (0.0005–0.5 µg/mL). This effect probably occurs through activation of the stress response and elevation of Hsp26p levels. The low effective dose holds therapeutic promise, as dactinomycin causes considerable side effects in oncology applications (Langholz et al. 2011). Finally, bioactive dietary compounds have been reported to modulate the solubility of huntingtin aggregates; notably, curcumin has been shown to interfere with the aggregation kinetics of HttEx1 in mammalian cells (Jain et al. 2025). Curcumin (a polyphenol present in the spice turmeric) decreases high molecular weight aggregates and increases lower molecular weight ones as observed in sucrose centrifugation gradients, and by fluorescence microscopy of Q72‐GFP (Verma et al. 2012). Curcumin prevents polyQ aggregate formation by reducing Vps36p expression. This protein is a component of the ESCRT‐II, the Endosomal Sorting Complex Required for Transport, and the endocytic pathway and vacuolar protein sorting, therefore essential to autophagy and the clearance of huntingtin aggregates (Meriin et al. 2002). Altogether, these works show how robust the yeast model is to find lead compounds that result in possible new treatment strategies for HD.

## 5 Spinal Cerebellar Ataxia (SCA)

SCA is a group of inherited, progressive neurological disorders characterized by the degeneration of the cerebellum and sometimes the spinal cord, leading to a progressive decline in coordination. Symptoms worsen over time and typically include: unsteady walking, stumbling, clumsiness, slow speech, involuntary eye movements, and difficulty swallowing. There are over 40 different numbered subtypes (e.g., SCA1, SCA2, SCA3, SCA6, etc.), each caused by a mutation in a different gene. Progressive polyQ expansion disorders include several SCA subtypes (SCA1, 2, 3, 6, 7, and 17).

SCA3, also known as Machado−Joseph disease, is caused by the expansion of a polyQ tract. Expression of toxic SCA3 polyQ expansions (e.g., 80Q) in yeast results in large cytoplasmic aggregates. Research shows the ubiquitin ligase Praja1 (PJA1) targets these aggregates for degradation, thereby suppressing polyQ‐mediated toxicity (Ghosh et al. 2021).

Spinocerebellar ataxia type 7 (SCA7) is caused by a polyQ expansion in the ATXN7 gene. Its yeast homolog, Sgf73, is a critical subunit of the SAGA and SLIK transcriptional coactivator complexes, which are essential for histone H3 and H2B acetylation and for maintaining complex integrity. Expression of a pathogenic polyQ‐expanded ATXN7 fragment (e.g., 60Q) in yeast disrupts these complexes, leading to altered composition and aberrant histone acetylation (McMahon et al. 2005). Furthermore, the stability of the yeast Sgf73 protein is regulated by the ubiquitin‐proteasome system, suggesting a potential quality control mechanism for the complex (Barman et al. 2023).

In contrast, SCA10 is caused by an abnormal pentanucleotide (ATTCT) repeat expansion within an intron of the ATXN10 gene. The pathogenic mechanism is distinct from polyQ disorders, and it is believed to involve RNA toxicity and impaired splicing. Yeast models have been instrumental in studying this instability. In one study, 46−81 copies of the ATTCT repeat were inserted into an artificial intron of the *URA3* reporter gene, preserving its function. However, expansions beyond approximately 85 repeats blocked *URA3* expression. This clever system enabled a genetic screen that identified key proteins—including Tof1, Rad5, and Rad52—that significantly influence the replication and repair of these unstable repeats (Cherng et al. 2011).

## 6 ALS

ALS is a neurodegenerative disease whose clinical manifestations in muscles include cramps, weakness, spasticity, and atrophy; it also causes respiratory insufficiency, difficulty in swallowing, difficulty with speech, cognitive and behavioral impairments (Brown and Al‐Chalabi 2017). Since cognitive and behavioral symptoms are observed in 50% of the patients (13% develop frontotemporal dementia), the initial motor neuron disease was redefined as a neurodegenerative disorder (Phukan et al. 2012).

### 6.1 ALS's Genetic Determinants

More than 30 genes are major risk factors of ALS, and cases of patients may show either a complex Mendelian inheritance pattern or no discernible family history. Four genes account for about 70% of all familial cases, to wit, C9orf72 (guanine nucleotide exchange C9orf72) (Brown and Al‐Chalabi 2017), FUS (RNA‐binding protein, fused in sarcoma) (Lagier‐Tourenne et al. 2010), SOD1 (superoxide dismutase 1) (Siddique et al. 1991), and TARDBP (TAR DNA‐binding protein 43) (Kabashi et al. 2008). As there are no published results on C9orf72 in *S. cerevisiae*, the following paragraphs present yeast ALS models used to understand the biology of FUS, SOD1, and TARDBP, related to the disease. While the primary cause of ALS remains unknown, a hallmark pathological feature involves the aggregation of FUS, SOD1, and TDP‐43 into cytoplasmic inclusions within motor neurons—a key mechanism implicated in disease pathogenesis.

### 6.2 FUS Model in Yeast

**Figure 4:**
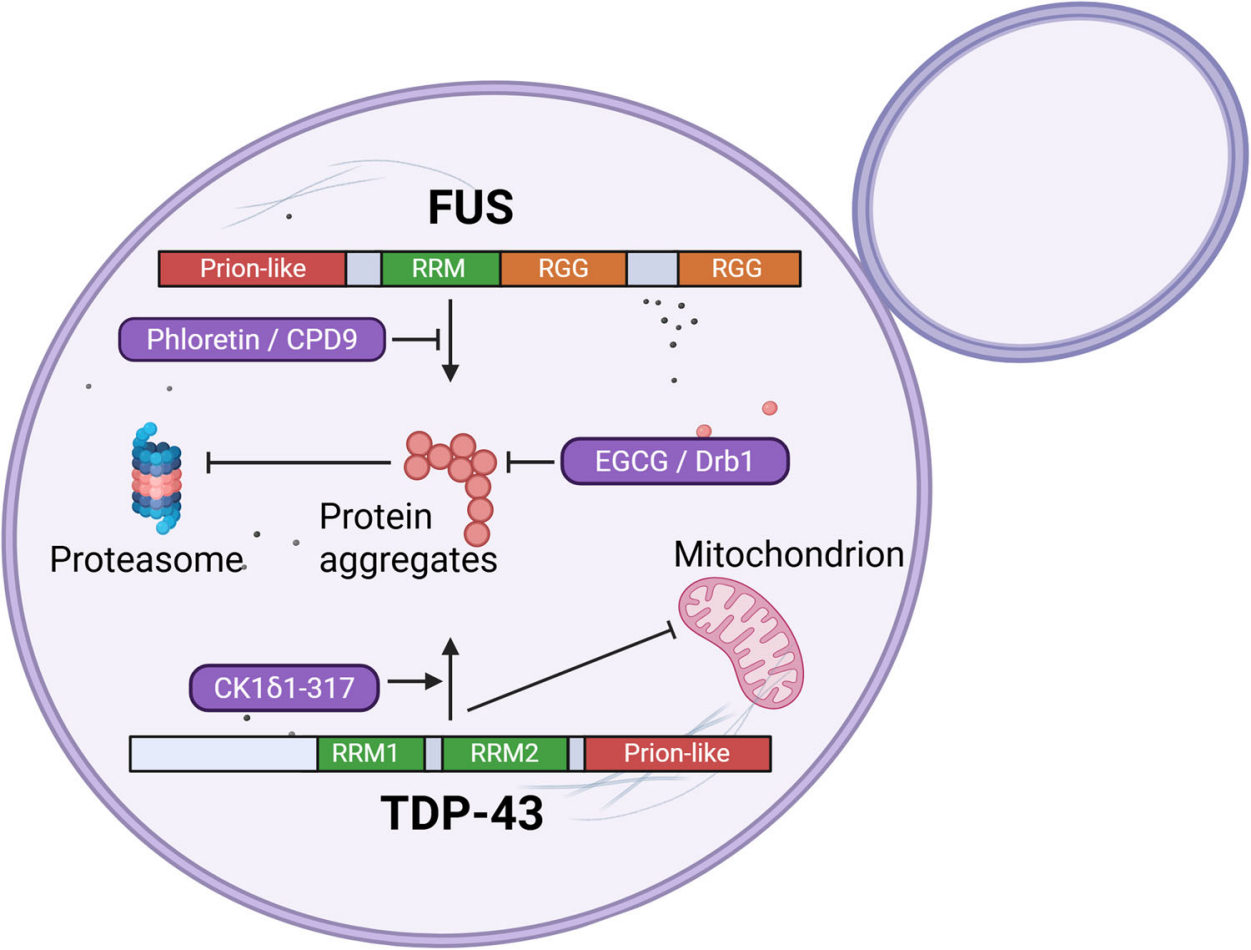
Yeast as a model of ALS disease. The FUS and TDP‐43 domains are depicted with the prion‐like region that promotes aggregation indicated in brown. Factors involved in the promotion or inhibition of the oligomer formation are also indicated.

In mutant forms, the RNA‐binding protein FUS mislocalizes from the nucleus to the cytoplasm. This mislocalization impairs its function, disrupting critical RNA processing mechanisms (Zhou et al. 2014). The FUS protein features an N‐terminal prion‐like domain, a glycine‐rich domain, and a C‐terminal region comprising one RNA recognition motif (RRM) followed by two Arg‐Gly‐Gly (RGG) repeats (Iko et al. 2004) (Figure 4). FUS‐GFP truncation constructs expressed in the yeast ALS model impair cell growth, and show that FUS determinants of aggregation are the first RGG repeat (aa 371–422) and the prion‐like domain (aa 1–239). However, FUS inclusions in yeast are mostly cytosolic, whereas in mammalian cells, nearly all the protein is diffuse and nuclear (Fushimi et al. 2011; Kryndushkin et al. 2011; Sun et al. 2011). Expression of many ALS‐linked FUS mutants, clustered at the C‐terminal region, identified in some familial and sporadic cases of the disease (Lagier‐Tourenne et al. 2010), shows a slight but not statistically significant reduction of FUS aggregates (Sun et al. 2011).

FUS contains phosphorylation sites at the prion‐like domain, which are a target of the DNA‐dependent protein kinase (DNA‐PK) (Deng et al. 2014). In yeast models, overexpression of human FUS leads to the formation of cytotoxic aggregates. Intriguingly, Sun et al. (2011) found that deleting a 25‐residue C‐terminal region—which harbors most ALS‐linked mutations—did not mitigate this aggregation. This finding appears paradoxical given that in mammalian systems, such as the FUS knock‐in mouse model (Sharma et al. 2016), the mislocalization of FUS from the nucleus to the cytoplasm is a critical step in ALS pathogenesis, a process often initiated by mutations in that very same region. Upon treatment of the FUS ALS yeast model with calicheamicin, double‐strand DNA breaks are formed and activate DNA‐PK, which results in a dramatic mobility shift of FUS protein on SDS‐PAGE gels, indicating its heavily phosphorylated status (Monahan et al. 2017). FUS phosphorylation sites analyzed from in vivo samples are found at S/TQ sites (target of DNA‐PK) pT7, pS26, pS30, pS42, and pS61, but also non‐S/TQ sites (pT71, pS77, and pS96). FUS phosphomimetics at these sites, or protein phosphorylation in vitro, prevent the formation of aggregates and show a more diffused protein in vivo, both in yeast and human cell lines. Altogether, these results indicate that by controlling FUS phosphorylation states, protein aggregation can be reduced in cells.

### 6.3 SOD1 Model in Yeast

Over 234 genetic variants of the cytosolic human Cu, Zn superoxide dismutase 1 (hSOD1) are associated with the risk of ALS (Benatar et al. 2025). The use of these alleles in the hSOD1 ALS yeast model shows that copper binding capacity and superoxide scavenging activity are properties equally maintained by all mutants that depend on the SOD1 copper chaperone Ccs1p for effective copper loading. Metal binding and an intramolecular disulfide bond stabilize the hSOD1 homodimer structure in the WT and ALS mutants, while loss of the intramolecular disulfide and formation of aberrant intermolecular disulfide aggregates the protein (Arnesano et al. 2004; Furukawa et al. 2006). Blocking copper activation in both Ccs1p and Ccs1p‐independent pathways severely alters the protein stability of ALS mutants A4V, G93A, and G37R but not WT (Carroll et al. 2006). The oxidation/dimerization of apo‐A4V hSOD1 in *grx1 grx2* mutants reveals GRX's critical role in suppressing aggregation of metal‐depleted ALS hSOD1 mutants. This relevance is further evidenced by age‐dependent accumulation of oxidized, high‐mass SOD1‐GFP inclusions in yeast cytoplasm (Brasil et al. 2019); evidence that oxidative damage is involved in the protein misfolding mechanisms observed in ALS and a clear connection to aging.

In mouse motor neurons overexpressing WT hSOD1, a fraction of this protein is secreted in exosomes. Similarly, yeast Sod1p can be extracted from the cell wall upon starvation in potassium acetate medium (Cruz‐Garcia et al. 2017). Under this condition, the sorting of yeast Sod1p to the extracellular compartment requires an essential diacidic motif E77 and E78. By introducing the mutation G93A in yeast SOD1, an ALS‐equivalent mutant is created whose cell wall extraction efficiency is similar to that of the WT protein. The mutant secretion is strongly impaired in triple mutant SOD1‐DE77/78AA/G93A that also lacks the acidic motif. These findings in the yeast ALS model are exceptionally provocative, since secretion of ALS mutants may contribute to the propagation of SOD1 toxicity along neuroanatomical pathways, and also activate microglia to induce noncell‐autonomous neurotoxicity (Grad et al. 2014).

The same approach was used to generate yeast SOD1 mutants that are equivalent to ALS A4V, G37R, H48Q, G93A, and S134N alleles. In the sod1 null mutant, not all alleles were expressed at the same levels during logarithmic growth, likely due to their inherent instability. In the WT SOD1 background, all isoforms were overexpressed and therefore became stabilized (Bastow et al. 2016). This is probably an effect of heterodimerization, which also increases the number of intracellular inclusions and raises lipoperoxidation and protein carbonylation levels (Brasil et al. 2019). Heterodimers of WT and ALS‐mutant SOD1 stabilize growth compared to *sod1* mutant cells, where ALS mutants impair budding and are toxic (Bastow et al. 2016). This restored growth enables cells expressing sod1A3V or sod1G92A to upregulate trehalose—a protective adaptation to aggregates, confirmed by metabolomic and enzymatic assays. Notably, trehalose exerts neuroprotection in Sod1G93A‐expressing mouse cells, likely by clearing aggregates via autophagy stimulation. The mechanism by which trehalose stimulates autophagy is species‐specific due to differences in metabolism. In mammalian cells, where trehalose is not metabolized, its cytosolic accumulation acts as an osmotic stressor that triggers autophagy, mitophagy, and reticulophagy (Xu et al. 2020). Conversely, in yeast, trehalose is a native carbon source and does not induce autophagy. Instead, the autophagic process is linked to trehalose catabolism, as deletion of *TPS2*—which encodes trehalose‐6‐phosphate phosphatase—lowers *ATG8* expression and inhibits autophagy (Kim et al. 2021).

Expression of hSOD1 ALS mutant A4V in *sod1* null mutant background shows an impact on the electron transport rates of complex III (70% reduction) and IV (55% reduction) when compared to the human WT protein (Gunther et al. 2004). Likewise, the mutant G93A reduced complex IV activity, but that of complex III was similar to the rate measured with mitochondria isolated from cells expressing WT hSOD1. In agreement with these data, heme content in A4V mutant cells shows an analogous decrease in hemes *b*, *c−c*_*1*_, and *a−a*_*3,*_ whereas G93A mutant shows a corresponding reduction in heme *a‐a*_*3*_content. Yeast Sod1p partitions at 0.7% from the cytosol to the mitochondrial intermembrane space (IMS). By fusing its gene with the cytochrome *b*_*2*_ bipartite mitochondrial targeting signal, b2‐Sod1p migrates exclusively to the IMS, where it improves cells' resistance to oxidants such as H_2_O_2_ and paraquat, strongly reducing protein carbonylation (Klöppel et al. 2010). ALS‐equivalent mutant *sod1* ^G93A^ partitions at 4% of the total Sod1p to the IMS, performing similarly to the b2‐Sod1p on the resistance to oxidants and protein carbonylation; however, the catalytically inactive *sod1* ^G85R^ shows the same localization distribution of the WT Sod1p but performs as poorly as control *sod1* null mutant cells. Surprisingly, the toxic effect of ALS‐equivalent mutants is not on mitochondrial function, but an impairment of the acidification of the vacuole (Bastow et al. 2016); the yeast organelle functionally analogous to the human lysosome, leading to autophagy reduction. Nevertheless, previous work shows that the loss of vacuole acidity leads to mitochondrial dysfunction that is linked to aging (Hughes and Gottschling 2012); therefore, it is necessary to conduct further investigation to clarify these conflicting results. A final illustration of the usefulness of the hSOD1 ALS yeast model concerns a yeast interaction trap system used to identify proteins interacting with ALS *sod1* ^*G93A*^(Kawamata et al. 2008). Lysyl‐tRNA‐synthetase (KARS) was found, and its interaction with the mutant was validated. KARS enzyme exists as two isoforms, one cytosolic (cytoKARS) and the other mitochondrial (mitoKARS). In mammalian cells expressing normal hSOD1 or the mutants G93A or G85R, there is preferential interaction between mitoKARS and ALS mutants (Kawamata et al. 2008), and that also occurs in the brain and spinal cord of transgenic mice. As a consequence of this interaction, KARS misfolds and aggregates, and a fraction of the protein is degraded by the ubiquitin‐proteasome system in the cytoplasm, where the other accumulates in the organelle. Eventually, this alters mitochondrial morphology, impairs mitochondrial DNA‐encoded protein synthesis, and leads to a decrease in survival.

The G93A mutation in SOD1 confers a tendency to misfold and aggregate. Within the endoplasmic reticulum (ER), these misfolded proteins disrupt proteostasis by overwhelming the ER's quality control systems, thereby triggering the UPR. This includes activation of the IRE1α pathway, evidenced by its unconventional splicing of XBP1 mRNA (Remondelli and Renna 2017).

### 6.4 TDP‐43 Model in Yeast

In many patients with ALS, the nuclear TAR DNA‐binding protein 43 (TDP‐43) is involved (Neumann et al. 2006). Mutations or high levels of TDP‐43 cause ALS, and in many patients, the protein is cleaved, hyperphosphorylated, and translocated to the cytosol, where it is ubiquitinated (Mitchell et al. 2015). TDP‐43 is an RNA‐binding protein whose mislocation affects the processing of its target RNAs, but also endosomal trafficking, particularly dendritic endosomes, which results in reduced neuronal signaling. Expression of TDP‐43 is toxic to *S. cerevisiae*. TDP‐43 recapitulates nuclear localization when the protein is expressed integrated as a single copy in the yeast genome, but that changes drastically to multiple cytoplasmic aggregates when expressed from a high‐copy (2 µ) plasmid (Johnson et al. 2008). The nature of this aggregation is surprisingly different because TDP‐43 foci are not diminished in the hsp101 mutant cell background like the huntingtin N‐terminal fragment, and, compared to the yeast prion [RNQ^+^], that forms SDS‐resistant high‐molecular‐weight amyloid‐like structures. Intriguingly, TDP‐43 has no amyloid‐forming ability, and it is not stained by thioflavin T or Congo red, two known amyloid‐binding dyes (Forman et al. 2007). The domain structure of TDP‐43 is analogous to FUS; it has an N‐terminal necessary for nuclear localization followed by two RRM motifs and a glycine‐rich C‐terminal prion‐like domain. In this structure, the determinants of protein aggregation are the second RRM motif followed by the C‐terminal prion‐like domain (Johnson et al. 2008). A set of TDP‐43 ALS‐linked mutations clusters in the protein's C‐terminal. When expressing these mutants from a low copy (CEN) plasmid, all of them are toxic and aggregate, some considerably more than the WT, and their expression causes cell death as indicated by propidium iodide staining (Johnson et al. 2008).

Unbiased genetic screenings to find modifiers of TDP‐43 toxicity in yeast found DBR1, which encodes an RNA lariat debranching enzyme, as a potent phenotype modifier, and knockdown of the DBR1 gene validates this in human neuronal cell lines and primary rat neurons (Armakola et al. 2012). Mutant DBR1 alleles lacking debranching function demonstrate that suppression of TDP‐43 toxicity requires Dbr1p enzymatic activity. The expression of mouse Dbr1 in yeast dbr1 mutant cells restores this capacity, indicating conservation from yeast to mammals. Since intronic lariats colocalize with TDP‐43 foci in yeast, the picture that develops from these findings is that in the absence of Dbr1p enzymatic activity, intronic lariats accumulate in the cytoplasm and sequester TDP‐43, preventing it from interfering with cellular functions. EGCG, first identified in yeast counteracting HTT oligomers, binds the tandem RRM of TDP‐43 and inhibits its aggregation (Morando et al. 2025). Another mammalian gene expressed in yeast causes TDP‐43 aggregation, and it is related to the observed phosphorylation of the protein. A hyperactive C‐terminal domain of the casein kinase 1δ (CK1δ1‐317) induces TDP‐43‐GFP cytotoxicity in yeast and aggregation also in human neuroblastoma cells (Figure 4) (Nonaka et al. 2016). This protein phosphorylates TDP‐43 at Ser‐409/410 residues, as revealed by immunoblotting. Furthermore, a recent genome‐wide yeast screen identified additional modifiers of TDP‐43 toxicity, including deletions of PBP1 and TIP41, which significantly alleviated TDP‐43‐induced cytotoxicity. These genes are involved in autophagy regulation, and their absence appears to enhance autophagic activity, suggesting that boosting autophagy may mitigate TDP‐43 proteotoxicity (Park et al. 2019). Taken together, the findings mentioned in this paragraph point out some possible interventions to reduce TDP‐43 toxicity in cells by either reducing Dbr1 or CK1 activity (Table 1).

**Table 1:** Neurodegenerative disease genes, yeast orthologs, and phenotypes associated with their total or partial expression in *Saccharomyces cerevisiae* cells.

Disease model	Gene	Phenotype in yeast	References
Alzheimer's	Tau No yeast ortholog	Conformation‐dependent antibodies detect pathological filament. Tau does not aggregate in yeast Yeast Mds1p (GSK3β). Pho85p (CDK5) is an ortholog of the major human tau kinases. In *pho85Δ* mutants, tau is hyperphosphorylated at the AD2 and PG5 epitopes. Oxidative stress triggers tau aggregation via a mechanism parallel to hyperphosphorylation.	De Vos et al. (2011) Vandebroek et al. (2005) Zubčić et al. (2024) Randez‐Gil et al. (2020) Vanhelmont et al. (2010)
	Pin1 Yeast ortholog: Ess1p	Ess1p isomerizes phosphorylated tau, preventing or reversing filament formation. Depletion of Ess1p results in tau hyperphosphorylation and impaired growth.	De Vos et al. (2011)
	PS1, Nicastrin, APH1, PEN2 No yeast orthologs	Co‐expression of human PS1, Nct, APH‐1, and PEN‐2 reconstitite γ‐secretase activity.	Yonemura et al. (2011) Imai et al. (2019)
	Aβ42	Aβ42‐GFP peptides aggregate in yeast cytosol and activate heat shock response Lower growth rate, lower biomass yield, lower respiratory rate, and increased oxidative stress.	Caine et al. (2007) Bagriantsev and Liebman (2006) Chen and Petranovic (2015) Treusch et al. (2011)
Parkinson's	SCNA (α‐synuclein) No yeast ortholog	Dose‐dependent toxicity. αsyn‐GFP forms aggregates that affect growth. Exacerbated mitochondrial dysfunction in the presence of menadione, mitigated by melatonin addition and mitophagy activation. Induces the formation of *petite* mutants and triggers programmed cell death. Affects vesicle trafficking and the endosomal network, oxidative stress, the unfolded protein response, calcineurin stimulation, protein degradation and autophagy, chaperone activity, and membrane composition.	Outeiro and Lindquist (2003) X. Zhang et al. (2024) Akintade and Chaudhuri (2020) Zampol and Barros (2018) Shadrina and Slominsky (2021) Menezes et al. (2015) Popova, Galka, et al. (2021)
	PARK8 (LRKK2, leucine‐rich repeat kinase 2) No yeast ortholog	PARK8 expression in yeast leads to growth arrest. Expression of PARK8 GTPase domain impairs endocytic vesicular trafficking, autophagy, and mitochondrial biogenesis.	Xiong et al. (2010) Aufschnaiter et al. (2018)
	PARK2 (Parkin) No yeast ortholog	PARK2 is directed to yeast mitochondria under oxidative stress to promote mitophagy.	Pereira et al. (2015)
	PARK7 (DJ‐1) Yeast orthologs: Hsp31p, Hsp32p, Hsp33p)	DJ‐1 overexpression reverses α‐synuclein toxicity, functionally complements *hsp31*Δ, and suppresses mitochondrial superoxide levels.	Zondler et al. (2014) Bankapalli et al. (2015)
	EIF4G1 (PARK18) Yeast orthologs: TIF4631 and TIF4632, encode for the translation initiation factor eIF4G VPS35 (PARK17) Yeast ortholog: Vps35p	Synthetic lethality of TIF4631 and VPS35 converged to the retromer complex (Vps26, Vps35, Vps29, Vps5, and Vps17), essential for α‐synuclein toxicity.	Dhungel et al. 2023 Simonetti and Cullen (2018)
	ATP13A2 (PARK9) Yeast ortholog: *YPK9*	Ypk9p vacuolar transporter protects cells from α‐synuclein cytotoxicity and divalent metal cations toxicity.	Gitler et al. (2009) Yeger‐Lotem et al. (2009)
Huntington's	HTT No yeast ortholog	Expression of 17 amino acids of the N‐terminal part, followed by the polyglutamine (polyQ) tract, and the proline‐rich domain fused to GFP forms aggregates in the cytosol (Q103). The Q25 protein does not aggregate. No aggregates are observed in *hsp104*Δ cells. Overexpression of *HSP104* increases the number of fluorescent foci, indicating dissolution of aggregates Q103 reduces oxygen consumption and diminishes complexes II and III activities of the mitochondrial respiratory chain, leading to elevated levels of ROS. Direct association between the polyQ tract and the mitochondrial outer membrane dissipates the mitochondrial membrane potential, decreases the energetic coupling, and impairs mitochondrial protein synthesis Q103 mitochondrial toxicity is reduced by the overexpression of HAP4.	Dehay and Bertolotti (2006) Krobitsch and Lindquist (2000) Meriin et al. (2002) Krobitsch and Lindquist (2000) Solans et al. (2006) Ocampo et al. (2010)
Spinocerebellar ataxia	ATXN3 No yeast ortholog	ATXN3‐polyQ expansions (80Q) result in large cytoplasmic aggregates Ubiquitin ligase Praja1 (PJA1) targets these aggregates for degradation.	Ghosh et al. (2021)
	ATXN7 Yeast ortholog: *SGF73*	Expression of ATXN7‐polyQ (60Q) disrupts SAGA and SLIK transcriptional coactivator complexes leading to altered composition and aberrant histone acetylation.	McMahon et al. (2005)
	ATXN10 No yeast ortholog	SCA10 is caused by a pentanucleotide (ATTCT) repeat expansion within an intron of the ATXN10 gene. 46−81 copies of the ATTCT repeat inserted into an artificial intron of the URA3 reporter gene, preserves its function, whereas beyond this point (over 85 repeats) blocked URA3 expression.	Cherng et al. (2011)
Amyotrophic lateral sclerosis	FUS	FUS‐GFP cytosolic inclusions impair cell growth. DNA double‐strand breaks activates the DNA‐dependent protein kinase (DNA‐PK), which results in FUS phosphorylation. FUS phosphomimetics prevent the formation of aggregates and show a more diffuse protein in vivo.	Fushimi et al. (2011) Kryndushkin et al. (2011) Sun et al. (2011) Monahan et al. (2017)
	SOD1 (human Cu, Zn superoxide dismutase 1) Yeast ortholog: *SOD1*	Blocking copper activation in both Ccs1p and Ccs1p‐independent pathways alters severely the protein stability of ALS mutants Sod1p‐A4V, ‐G93A, and ‐G37R but not WT. Age‐dependent accumulation of oxidized, high‐mass SOD1‐GFP inclusions in yeast cytoplasm. Yeast Sod1p can be extracted from cell wall upon starvation in potassium acetate medium, in a mechanism related to SOD1 prion in humans. SOD1 mutants impair budding and are toxic. hSOD1 ALS mutant A4V in *sod1*Δ background impacts on the electron transport rates of complex III and IV.	Carroll et al. (2006) Brasil et al. (2019) Cruz‐Garcia et al. (2017) Bastow et al. (2016) Gunther et al. (2004)
	TDP43 No yeast ortholog	TDP‐43 expressed in a single copy does not aggregate, whereas multiple cytoplasmic aggregates are observed when expressed from a high‐copy (2 µ) plasmid. TDP‐43 foci are not diminished in hsp101 mutant cells background like huntingtin N‐terminal fragment. The determinants of protein aggregation are the second RRM motif followed by the C‐terminal prion‐like domain. A set of TDP‐43 ALS‐linked mutations clusters to the protein C‐terminal. When expressing these mutants from a low‐copy (CEN) plasmid, all of them are toxic and aggregate, and their expression causes cell death. Cells lacking DBR1, an RNA lariat debranching enzyme, show suppression of TDP‐43 toxicity. Deletions of PBP1 and TIP41, genes that negatively regulate autophagy, significantly alleviate TDP‐43‐induced cytotoxicity. TDP‐43 is more toxic in respiring cells.	Johnson et al. (2008) Morando et al. (2025) Park et al. (2019)

In mammalian cells, TDP‐43 is localized to the mitochondria and promotes mitophagy (Hong et al. 2012). In the mouse model, the amount of this localization is increased by TDP‐43 ALS mutations, and it is associated with mitochondrial dysfunction (Y.‐H. Wang, Li, et al. 2013; W. Wang et al. 2016). When expressed in yeast, TDP‐43 does not affect the growth of cells on glucose medium (fermentation) but inhibits the growth on galactose (a condition where respiration and fermentation are present), and more severely, the growth on ethanol or glycerol media, that promotes exclusively respiration (Park et al. 2019). Although TDP‐43 aggregates in all media tested, it causes more aggregation on galactose and glycerol, and an elongation phenotype of the cells is also observed (Park et al. 2019). This shows that TDP‐43 is more toxic in respiring cells, and may pave the way to find the TDP‐43 ALS mutants or structural determinants that, respectively, exacerbate or are linked to the phenotype. In summary, hSOD1 and TDP‐43 ALS models revealed important aspects of these proteins related to mitochondrial function, indicating cellular conditions that promote neurodegeneration in ALS, which can be further studied to identify targets for small‐molecule screens. Alternatively, although absent in metazoans, the disassembler protein Hsp104 effectively removes α‐synuclein, TDP‐43, and FUS aggregates in yeast. Engineered versions with increased aggregate specificity were developed to create therapeutics for neurodegenerative diseases. (Mack et al. 2023).

## 7 Conclusions

Protein misfolding and trafficking dysfunction are primary drivers of pathology in neurodegenerative diseases. Studying these inherently complex processes in neurons is challenging, but simpler cellular models like yeast offer a powerful alternative.

Key mechanisms are shared between yeast and neurons: chaperones in neurons and yeast interact with highly reactive proteins in their folding process, preventing inappropriate interactions during folding; protein remodeling factors can prevent and reverse protein aggregation; the proteasome degrades by proteolysis damaged or misfolded proteins; and in autophagic processes, cells remove unnecessary or dysfunctional components, including entire organelles. Vesicular trafficking is also conserved. In yeast cells, the secretory pathway and vesicular trafficking are responsible for molecular traffic between specific membrane‐enclosed compartments around the cell in a highly orchestrated way for mating pheromone transport, receptor trafficking, and cell wall components deposition. Likewise, neuronal vesicle trafficking manages neurotransmitters, neurotrophic factors, and extracellular matrix deposition, creating a microenvironment that modulates neuron function. Neurons and yeast have the same organelles, including RE, Golgi, lysosomes, peroxisomes, and mitochondria. Both cell types share quality control pathways, including autophagy and apoptosis, and signaling transduction pathways that have been preserved during evolution, including AMPK, calcineurin, and TOR (Dhakal and Macreadie 2020). Therefore, it is possible to model the unique, multifaceted toxicities of different proteins at the cellular level using yeast, but it requires a careful experimental design considering the slow development of neurodegenerative proteotoxicity during aging (Ruetenik and Barrientos 2018; Jiang and MacNeil 2023; Stella et al. 2025).

Altogether, the studies reported here demonstrate significant advances achieved using yeast as a model organism to address fundamental problems directly related to neurodegenerative disease development. The study of important amyloidogenic disease‐causing proteins in yeast leads to important discoveries about the metabolism and toxicity of such proteins in a simpler cell model, allowing the identification of partners and new factors involved in each pathway; more definitely, the identification of chemicals and druggable pathways provides targets of natural compounds or small molecules that can be a potential treatment for each disease. Since yeast cells can be humanized way beyond the expression of only single aggregation‐prone proteins, complex cellular dynamics may be studied when more human factors are added, to understand the biological problem in conditions that recapitulate the pathological events in neurons.

## Author Contributions

J.R.F.J., V.L.C., and M.H.B. conceived and drafted the manuscript as well as prepared the digital images.

## Data Availability

Data sharing is not applicable to this article as no data sets were generated or analyzed during the current study.
